# A Hybrid Neural Network Model for Sales Forecasting Based on ARIMA and Search Popularity of Article Titles

**DOI:** 10.1155/2016/9656453

**Published:** 2016-05-22

**Authors:** Hani Omar, Van Hai Hoang, Duen-Ren Liu

**Affiliations:** ^1^Institute of Information Management, National Chiao Tung University, Hsinchu 300, Taiwan; ^2^Computational Intelligence Technology Center, Industrial Technology Research Institute, Chutung, Hsinchu 310, Taiwan; ^3^The University of Danang, Campus in Kon Tum, No. 129 Phan Dinh Phung Street, Kon Tum 580000, Vietnam

## Abstract

Enhancing sales and operations planning through forecasting analysis and business intelligence is demanded in many industries and enterprises. Publishing industries usually pick attractive titles and headlines for their stories to increase sales, since popular article titles and headlines can attract readers to buy magazines. In this paper, information retrieval techniques are adopted to extract words from article titles. The popularity measures of article titles are then analyzed by using the search indexes obtained from Google search engine. Backpropagation Neural Networks (BPNNs) have successfully been used to develop prediction models for sales forecasting. In this study, we propose a novel hybrid neural network model for sales forecasting based on the prediction result of time series forecasting and the popularity of article titles. The proposed model uses the historical sales data, popularity of article titles, and the prediction result of a time series, Autoregressive Integrated Moving Average (ARIMA) forecasting method to learn a BPNN-based forecasting model. Our proposed forecasting model is experimentally evaluated by comparing with conventional sales prediction techniques. The experimental result shows that our proposed forecasting method outperforms conventional techniques which do not consider the popularity of title words.

## 1. Introduction

Forecasting is an important part of many aspects of our lives, and sales forecasting plays a major role for enterprises in making business plans more accurate and gaining competitive advantage. Enhancing sales and operations planning through forecasting analysis and business intelligence is demanded in any industry and business. Convenience stores allow people to buy things at anytime and anywhere. They have a huge market in Taiwan and many publishing providers (e.g., magazine business) want to cooperate with them. Magazine businesses need plans for enhancing sales, distribution, storage space, and high quality predictions. Accurate sales forecasting can reduce inventory costs and shortages. It would increase profits for the business by reducing wasted resources and allow planning for appropriate future production.

Most studies have depended on historical sales data for forecasting sales, but the sales of magazines are also affected by the contents of the magazines. The contents are represented by the stories (articles) and their titles. Popular content can often boost sales, so it is an important field to consider when forecasting sales. For example, a magazine reporting celebrity gossip, for example, Faye Wong's extramarital affair, will attract readers who like her and want to know about her to buy the magazine. Thus, magazine sales will increase due to the popularity of the magazine contents. Before customers make decisions, they usually search for a product via the Internet in websites and blogs. Thus, search indexes of product data received via the Internet can be useful in deriving the popularity of products for building prediction models. Search terms related to the products can be decided manually. However, the terms manually decided may be limited when approximating consumer preferences. In this paper, information retrieval techniques are adopted to extract words from article titles. The popularity measures of article titles are then analyzed by using the search indexes obtained from Google search engine. The derived popularity denotes consumer interests in the contents of the magazines. The higher values of search indexes from the Google search engine indicate higher popularity and interest in magazine contents.

Several forecasting models, such as regression models [1–3], exponential smoothing and Box-Jenkins models [4–7], neural networks [8–12], or fuzzy system models [13–15], have been developed and implemented in different sectors of business. Many researchers have achieved accurate sales forecasting by using a time series method such as the autoregression (AR), moving average (MA), and autoregressive moving average (ARMA). Previous studies used statistical methods to make the trends stable. These methods found the maximum likelihood fitting to be ARMA [5]. The major limitation is the preassumed linear form of the models of AR, MA, and ARIMA. That is, a linear correlation structure is assumed among the time series values; no nonlinear patterns can be captured by the ARIMA model [16].

Time series are generally recognized as nonlinear and/or nonstationary. Due to these two properties, the system should be sensitive enough to capture the uniqueness of a time series and should replicate this in prediction. Traditional time series methods are confined to the assumption of linearity; however, some data are nonlinear. In order to overcome the limitations of the traditional methods, many researchers are using soft computing techniques such as fuzzy logic, neural networks, fuzzy neural networks, and evolutionary algorithms.

Neural networks are reported to be such a method. Artificial neural networks (ANNs) have more noise tolerance than conventional regression-based empirical modeling, having the ability to learn complex systems with incomplete and corrupted data. In addition, they are more flexible, having the capability of learning dynamic systems through a retraining process using new data patterns with proper training data set [17, 18]. Forecasting reliability of the proposed neural networks was expressed in terms of forecasting error performance based on statistical and graphical methods. The most common learning mechanism is the “backpropagation” approach. The Backpropagation Neural Network (BPNN) is a supervised learning network well suited for prediction [19, 20].

In our preliminary work [21], we have proposed a BPNN popularity method, a sales forecasting model based on Backpropagation Neural Network (BPNN) where the inputs are historical sales and the popularity indexes of magazine article titles. In this study, we extend and enhance our preliminary work by proposing a novel hybrid ARIMA-BPNN popularity model for sales forecasting based on the prediction result of time series forecasting and the popularity of article titles. The proposed hybrid model uses the historical sales data, popularity of article titles, and the prediction result of a time series, Autoregressive Integrated Moving Average (ARIMA) forecasting method to learn a BPNN-based forecasting model. More specifically, our proposed BPNN model considers not only historical sales data but also the popularity of article titles. Besides, our proposed hybrid model uses the prediction output of ARIMA as input for the BPNN and uses the BPNN for forecasting future sales. The motivation for the hybrid model comes from the following perspectives. First, it is often difficult in practice to determine whether a time series data under study is generated from a linear or nonlinear underlying process or whether one particular method is more effective than the other in forecasting. Second, real-world time series are rarely purely linear or nonlinear. They often contain both linear and nonlinear patterns. The ARIMA model cannot deal with nonlinear relationships while the neural network model alone is not able to handle both linear and nonlinear patterns effectively. Several empirical studies have also suggested that combining several different models can improve forecasting accuracy [16, 22]. However, existing studies have not considered the effects of content popularity on sales. Our proposed hybrid BPNN model considers the popularity of article titles and prediction output of ARIMA can be a more effective approach for sales forecasting.

Our proposed forecasting model is experimentally evaluated by comparing with conventional sales prediction techniques. The experimental result shows that our proposed forecasting method outperforms conventional techniques which do not consider the popularity of title words. Our proposed hybrid ARIMA-BPNN popularity method also outperforms the BPNN popularity method presented in our preliminary work.

Section 2 provides related work on the methodology of forecasting.  Section 3 describes a proposed hybrid methodology for forecasting sales. The experimental results and evaluation of our dataset using our proposed models are in Section 4.  Section 5 provides a discussion of the results and analysis of real data supplied from a Taiwanese magazine. The conclusion is in Section 6.

## 2. Related Work

There are many forecasting methods, and they are very important for managers and businessmen. Forecasting allows them to anticipate future operating expenses, enables more confident strategic decisions, and increases business efficiency. Additionally, it can be applied to identifying key areas in a business operation that operators can influence proactively. Therefore, developing AI systems for this kind of prediction is not simple [23]. There are two types of forecasting, traditional and nontraditional forecasting methods.

### 2.1. Traditional Forecasting Methods

These techniques are easy to develop and implement. Traditional methods include time series regression, moving average (MA), Autoregressive Integrated Moving Average (ARIMA), and exponential smoothing [24, 25]. ARIMA is a popular traditional method used to analyze stationary univariate time series data. The main idea of ARIMA is to build a mathematical model with historical data to represent the regular pattern of a time series. Then, using this model and historical values, forecast the future values of this time series. There are usually three main stages to building an ARIMA model, including model identification, model estimation, and model checking. Model identification is the most crucial stage in building ARIMA models [26]. ARIMA is the method introduced by Box-Jenkins and has been used in various fields [1] and this model is a great predictor in the linear modeling problems but has poor performance for nonlinear problems. The ARIMA model is a generalization of the ARMA model, which integrates an autoregression (AR) and moving average (MA). Regression analysis is a method of investigating the relationships between variables, while moving average is a time series constructed by taking the averages of several sequential values of another time series.

### 2.2. Nontraditional Forecasting Methods

**Figure 1:**
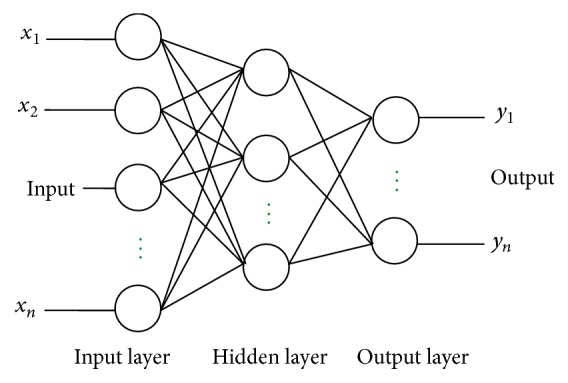
Neural network model.

A number of researchers have developed nonlinear forecasting models for predicting stocks or sales [27]. Neural networks (NN) represent the nonlinear forecasting model and can be applied to time series modeling without assuming a priori function forms of models to find the nonlinear relationship between inputs and outputs [28]. Traditional time series methods may not be able to capture nonlinear patterns in data. NN modeling is a promising alternative for overcoming these limitations. Neural processors are designed based on cross correlation between inputs and the weights [29]. For instance, when training a neural network, the expected and real outputs of each sample of training set are compared, and the error information is used to modify the weights and thresholds [30, 31]. In a feed-forward network, there can be several hidden layers, with each layer having one or more nodes. The number of hidden layers and the number of nodes in each hidden layer are usually determined by a trial-and-error procedure [32]. A type of ANN, the multilayer perceptron trained by a backpropagation algorithm, has been utilized in many fields for forecasting. When compared to other traditional methodologies the backpropagation procedure is one of the most appropriate procedures for forecasting, especially for nonlinear data. Backpropagation is updating the weights in each perceptron to justify the real results as matching or approaching the desired result.  Figure 1 shows a model of a neural network.

Each model may have shortages related to the model itself or related to data. Thus, combining different models as hybrid models is used to overcome these shortages or enhance the performance. For example, an ANN predictor may give more accurate load forecasts during the morning hours, while a linear regression predictor may be superior for evening hours. Hence, a method that combines different types of predictors may outperform any single type of predictors [33]. Furthermore, weighted combination methods can increase the prediction accuracy by assigning greater importance to predictors of higher credit [34]. Traditional statistical models such as Autoregressive Integrated Moving Average (ARIMA) are linear prediction models. These models have been constrained to using linear functions in past observations. Several classes of nonlinear models have been proposed to overcome the restrictions of the linear models and account for certain nonlinear situations. ARIMA and ANN models have achieved successes in both linear and nonlinear domains [16]. Areekul et al.'s [35] hybrid model depends on using the ARIMA and ANN jointly. Their model used the ARIMA as a forecasting method for the linear component of the time series data and used ANN to forecast the residual as a nonlinear component. They combined the predicted values from the time data series and its residuals to improve forecasting accuracy.

## 3. The Hybrid Methodology Based on the Popularity of Article Title Words

Popular article titles and headlines attract readers to buy the magazine and play a major role in sales. In this paper, we use the popularity of words in titles and headlines to improve the accuracy of forecasting. Information retrieval techniques are adopted to extract words from article titles. We then analyze the contents (title words) of magazine articles to measure the popularity of magazines based on the search indexes of title words derived from Google search engine. We propose forecasting with the Backpropagation Neural Network with popularity (BPNN-POP). Also, we propose a hybrid (Hybrid-POP) model with popularity combining the BPNN-POP and Autoregressive Integrated Moving Average (ARIMA) using popularity.

**Figure 2:**
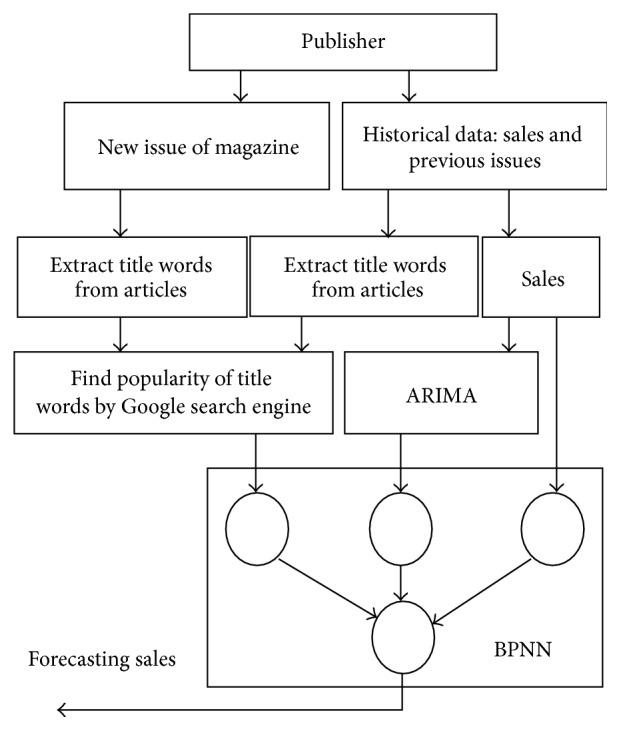
Hybrid ARIMA-BPNN popularity model.

Our proposed forecasting models have preprocesses, starting with finding the correlation of sales, extracting the title words (phrases) from our data, finding the popularity of a magazine's content, and normalizing both the popularity and historical sales data. Our model (Hybrid-POP) uses historical sales data as input for an ARIMA. We then use the predicted value from the ARIMA and the historical sales data as input for the BPNN-POP, which uses popularity for forecasting sales. Therefore, a pattern of linear data and nonlinear data time series can be captured (Hybrid-POP).  Figure 2 depicts our model (Hybrid-POP) with Autoregressive Integrated Moving Average and Backpropagation Neural Network with popularity.

### 3.1. Popularity Analysis of Magazine Content

Sales of publications such as magazines may be influenced by factors including content, social stratification, age, gender, locations of outlets, and historical sales data. In this research, we concern ourselves with historical sales data and the contents of a magazine. Historical sales data is commonly used as input for most forecasting models, but there is a need to normalize sales by dividing the sales over the maximum of all sales of magazine issues. Popular content can usually attract consumer interest and boost magazine sales. Therefore, we extract title words from magazine articles to obtain title word search indexes (volumes) using Google search. There are different ways to judge popularity. Google is the largest web search engine company; its search service is very powerful and useful.

**Figure 3:**
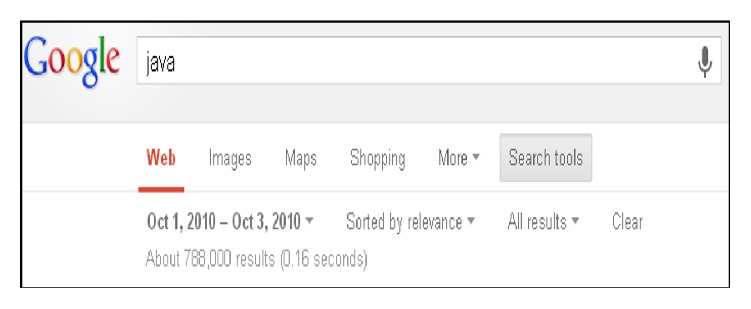
Using Google search.

Information retrieval techniques are adopted to extract words from article titles. The popularity of a title word can be derived from the Google engine to determine how many webpage volumes they have related to the title word. The Google engine also can restrict results to a particular time frame, (e.g., from October 1st, 2010, to October 3rd, 2010), and the results can show the number of retrieved webpages related to the title word in a time window. Generally, more retrieved webpages for the title word indicate higher popularity.  Figure 3 shows an example of using a Google search engine to find the volume of usage for the word “java”. The search engine is used to derive the popularity scores of title words by finding the search indexes, that is, the number of webpages for each title word during a one-month search period before the publication date of each issue. We accumulate the popularity scores of all title words for each issue and use them to represent the popularity index of the issue:

(1) $$\begin{array}{c} P_{t}=\overset{N_{t}}{\underset{j=1}{\sum }}R_{t,j}, \end{array}$$

where *P*_*t*_ is the popularity index of issue *t*; *N*_*t*_ is the number of title words in issue *t*; and *R*_*t*,*j*_ is the outcome of the Google search for title word *j* in issue *t*. The popularity indexes are normalized by dividing the maximum value over all issues.

### 3.2. Autoregressive Integrated Moving Average (ARIMA)

ARIMA has been one of the popular linear models for time series forecasting during the past three decades [16]. In this research, we aimed to use it as part of our adopted hybrid forecasting model and as a benchmark for testing the effectiveness of our proposed model. The ARIMA model is nonseasonal and has parameters. In the ARIMA(*p*, *d*, *q*) model, (*p*) is the number of autoregressive terms; (*d*) is the number of nonseasonal differences; and (*q*) is the number of lagged forecast errors in the prediction equation [36]. ARIMA has the following model:

(2) 1−φ1L−φ2L2−⋯−φpLp1−Ldyt=c+1−ψ1L−ψ2L2−⋯−ψqLqεt,

where *φ*_*i*_  (*i* = 1,2,…, *p*) and *ψ*_*j*_  (*j* = 1,2,…, *q*) are the model parameters. *p* is an order of autoregressiveness, and *q* is an order of moving average part while *d* represents order differencing. *ε*_*t*_ is white noise and is assumed to be independently and identically distributed with a mean of zero and a constant variance of *σ* ^2^. ARIMA was developed as a practical approach [37]. This methodology includes three iterative steps of model identification, parameter estimation, and diagnostic checking.

In the identification step, remaining stationary is necessary for building an ARIMA model. Therefore, data series have statistical characteristics such as a mean and variance that are constant over time. Parameters are estimated linearly when the model is specified, and nonlinear data series are estimated by minimizing the measure of errors. The last step is basically to check if model assumptions about the errors *ε*_*t*_ are satisfied. If the model is not adequate, a new model should be identified, which is again followed by the steps of parameter estimation and model verification [16].

### 3.3. Backpropagation Neural Network with Popularity

BPNN is a common method of teaching artificial neural networks how to perform a given task. It is a supervised learning method, consisting of a network system containing a large number of simple processing elements which are fully interconnected. In order to make the actual output close to any complex nonlinear mapping, its information processing procedure includes backpropagation, forward propagation, and weight adjustment. BPNNs are a type of feed-forward artificial neural networks. Feed-forward neural networks consist of an input layer, a hidden layer, and an output layer, as depicted in Figure 2. Each layer is formed by a number of nodes, and each node represents a neuron. The data flow in one direction and answers are obtained solely based on the current set of inputs. A BPNN has the same structure as a forward artificial network but is trained with a backpropagation algorithm in which incidences of training samples are entered from the input layer, and the outputs are calculated through the operation of corresponding functions and connection weights between the nodes [38]. The output error is obtained by comparing the calculated values with the target outputs. Sigmoid function is usually used as the activation function in the hidden layer of backpropagation:

(3) $$\begin{array}{c} f\left(\;I\;\right)=\frac{1}{1-e^{-I}}, \end{array}$$

where *I* is the next formula and

(4) $$\begin{array}{c} I=\overset{n}{\underset{j=1}{\sum }}w_{mn}x_{n}, \end{array}$$

where *w*_*mn*_  (*i* = 0,1,…, *m*; *j* = 1,2,…, *n*) are the connection weights, *m* is the number of hidden nodes, *n* is the number of input nodes, and *x* is the input value. We used a metric to calculate the difference between the real sales and the predicted values. There are many metrics, but one of them is enough to use for adjusting the (interconnections) weights in the neural network model.

**Figure 4:**
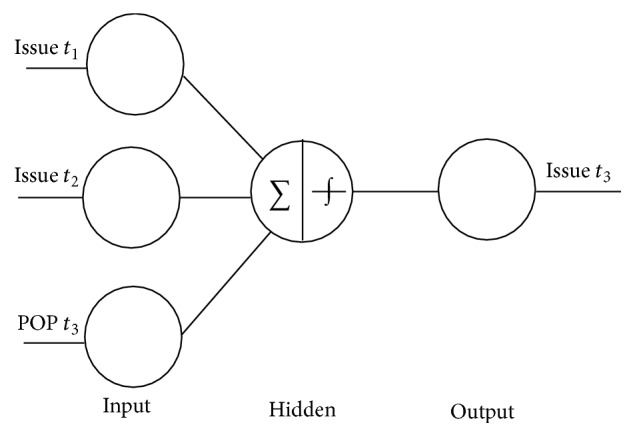
Three inputs for the BPNN-POP.

In our proposed model of the Backpropagation Neural Network with popularity (BPNN-POP), we used three inputs; two inputs represented the sales of the previous two issues (Issue *t*_1_ and Issue *t*_2_) before the target issue (Issue *t*_3_) and the third input represented the popularity of article titles and headlines for the target issue.  Figure 4 demonstrates a BPNN-POP with three inputs, including previous sales of the last two issues. The third input is the normalized popularity (POP *t*_3_) of the target issue.

### 3.4. Hybrid ARIMA-BPNN Popularity Model

**Figure 5:**
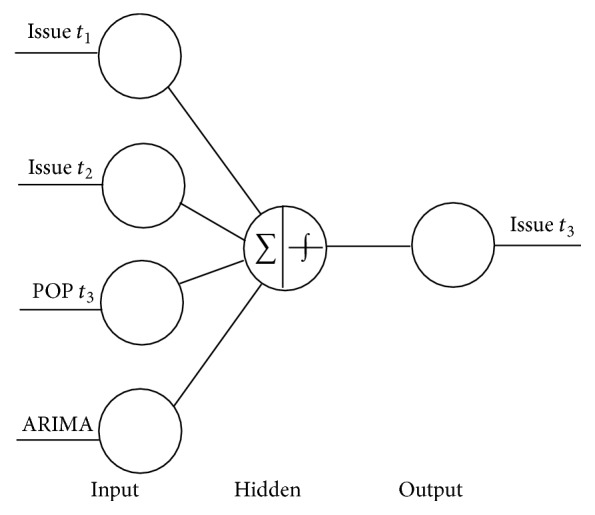
Four inputs for the hybrid ARIMA-BPNN popularity model (Hybrid-POP).

Our proposed model adopts the hybrid model (Hybrid-POP) for forecasting sales. In the hybridization, the ARIMA is used to forecast sales of a target issue by using the sales of previous two issues. Predicted sales from the ARIMA are used as input for the BPNN-POP along with sales from the previous two issues and the popularity of the target issue. The implementation of Hybrid-POP has four inputs, as shown in Figure 5. The first two inputs represent the sales of the previous two issues (Issue *t*_1_ and Issue *t*_2_) before the target issue (Issue *t*_3_) for forecasting. The third input represents the normalized popularity of the target issue. The fourth input represents the forecasting value of the target issue using ARIMA. Using the univariate ARIMA model has constraints. ARIMA is not tolerant of any extreme values in data and should be reestimated after every new data point. BPNNs are more tolerant of extreme values in data as compared to the ARIMA. Another constraint is the nature of data as being either linear or nonlinear. For these reasons, we use the hybrid model (Hybrid-POP) combining the ARIMA and the Backpropagation Neural Network using popularity to predict sales of a target issue. In the first step of our hybrid system, an ARIMA model is used to model the linear part of the time series and to create the ARIMA forecast. In the next step, the outputs of ARIMA are used as inputs for the artificial neural network and trained using known input and output training data to model the nonlinearity part of the time series. This method emphasizes the effects of previous sales and the popularity of article titles in the BPNN's forecasting method as well as the effect of the ARIMA by using the predicted value from it as an input to the neural network. The Hybrid-POP can thus tolerate more extreme values without reestimating the process. Moreover, attractive titles and headlines in magazines have an effect on buyer preferences for these publications. The proposed Hybrid-POP uses the popularity of article titles to enhance the accuracy of forecasting.

## 4. Experiment and Evaluation

In this section, we conduct experiments on our forecasting method and other methods and compare them to find out what we can achieve by using the popularity of magazine contents in our adopted hybrid method for forecasting, as described in Section 3.

### 4.1. Experiment Set-Up

In this section, we describe our sales forecasting model utilizing ARIMA, Backpropagation Neural Networks (BPNNs), and the popularity indexes of magazine article titles. We implemented our hybrid forecasting model using sales data from a Chinese magazine categorized as a weekly magazine. The data set includes article titles from 133 weekly issues sold through stores from June 2009 to December 2011. We chose the 50 stores with the highest correlations of sales and popularity indexes of magazine issues. We used the first 80 weeks of sales for each of the top 50 stores as the training set and the remaining 53 weeks of sales as the testing set.

Extracting the words from the titles and headlines of magazine is part of the preprocessing. We used the Google search engine to derive popularity scores of the extracted words of titles for each issue during a specific time period. We used one month as a period time to determine popularity. Note that the store sales (the numbers of sold items) are normalized to derive the normalized numbers of sold items during the processing of various forecasting methods. We normalized the store sales and popularity scores by dividing the store sales over the maximum of all store sales and the popularity scores over the maximum of all popularity scores, respectively. The following equation shows how to normalize the store sale (the number of sold items) for each issue (weekly issue):

(5) $$\begin{array}{c} Normalize\left(\;\#\;\;item_{t}\;\right)=\frac{\#\;\;item_{t}}{Max\left(\#\;\;item\right)}, \end{array}$$

where #  item_*t*_ is the number of sold items for issue *t* and Max(#  item) is the maximum number of sold items in stores. The normalization of popularity scores is derived similarly.

#### 4.1.1. Evaluation Metrics

The forecasting quality depends on the residuals of the actual number of sold items for each issue and the predicted number of items for that issue. There are many measurements to evaluate the residuals. We used the Root Mean Square Error (RMSE), which evaluates the average residuals for predicted sales (the numbers of sold items) as defined in the following:

(6) $$\begin{array}{c} RMSE=\sqrt{\frac{1}{N}\overset{N}{\underset{t=1}{\sum }}{\left(Sales_{t}-prediction_{t}\right)}^{2}}, \end{array}$$

where *N* is the number of issues in the testing set, Sales_*t*_ is the actual number of sold items for issue *t*, and prediction_*t*_ is the predicted number of items for issue *t* by the forecasting method. The magazine issue is published weekly. We also used RMSE in the BPNN in the learning step. The network “learns” by adjusting the interconnections (called weights) between layers in the BPNN. Thus, the RMSE is an appropriate evaluation metric in this work for adjusting the weights in the BPNN and evaluating our hybrid method in comparison to BPNN and ARIMA. We should note that RMSE could also be calculated based on the normalized values of Sales_*t*_ and prediction_*t*_ by using (5).

#### 4.1.2. Methods Compared in the Experiment

In the forecasting field, many methods have been used and there is no definitive claim for the most accurate forecasting method for any data time series. Comparison is a way to see the more accurate forecasting method for our data set. We used the Autoregressive Integrated Moving Average (ARIMA) and the Backpropagation Neural Network (BPNN) without popularity on titles and headlines as benchmarks. Mean Root Square Error (MRSE) was used as a metric to estimate the improvement of the accuracy of our Backpropagation Neural Network with popularity (BPNN-POP) and our hybridization of ARIMA and BPNN-POP (Hybrid-POP) when comparing with other methods. We use multilayer perceptron function from WEKA 3.6.13 to implement BPNN with the following values: the learning rate is 0.1, the momentum is 0.2, decay is False, seed is 0, and training time is 500 epochs. The computational cost of BPNN for each epoch requires *O*(*N* × *w*) time, where *N* is the number of training samples and *w* is the number of weights in the neural network. For 500 epochs, the computational cost of BPNN is approximate to *O*(*N* ^2^). The computational cost of ARIMA is linear time *O*(*N*). The hybrid model (Hybrid-POP) used the historical number of sold items, popularity, and the predicted number of items from the ARIMA forecasting model as inputs. The computational cost of the hybrid model also depends on the BPNN model. Accordingly, the computational cost of the Hybrid-POP is *O*(*N* ^2^).

**Figure 6:**
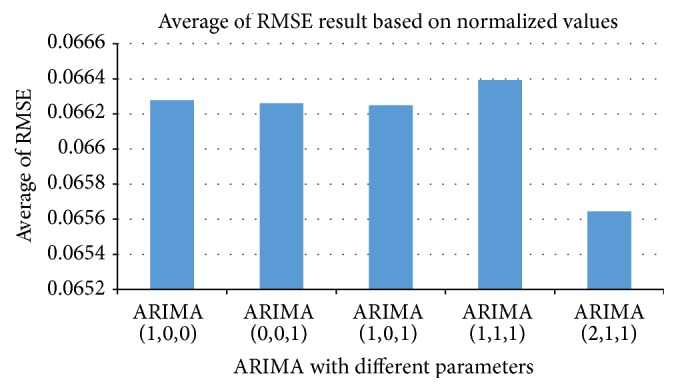
Average of RMSE (by normalized values) for ARIMA(*p*, *i*, *q*) with different values of parameters.

*Autoregressive Integrated Moving Average (ARIMA).* We used the number of sold items of the previous two issues to forecast the target issue using the ARIMA model. We assumed five different parameters for the ARIMA to identify the model. We randomly select 10 stores to conduct experiments on identifying the best parameters for the ARIMA model.  Figure 6 shows the average RMSE for sample of our data set for each of the following: ARIMA(1,0, 0), ARIMA(0,0, 1), ARIMA(1,0, 1), ARIMA(1,1, 1), and ARIMA(2,1, 1). Note that the RMSE of each store is calculated based on the normalized values of the actual numbers of sold items and predicted numbers of items. As seen in Figure 6, ARIMA(2,1, 1) is more appropriate than the others. Therefore, we used it as one of our methods. We used the output of ARIMA(2,1, 1) as an input for BPNN in our Hybrid-POP method to capture the linear and nonlinear patterns of our time data series for each issue of the magazine.

*Backpropagation Neural Network without Popularity (BPNN)*. The Backpropagation Neural Network was used as the forecasting model for nonlinear data. This is the typical forecasting method, which depends on historical number of sold items data. We used the number of sold items of the previous two issues of our target issue as input for the BPNN. We used the RMSE for measurement and for adjusting the interconnections. In this model, we used one hidden layer. After using the training set in the BPNN, the testing set was used to get the predicted values from two models.

*Backpropagation Neural Network with Popularity (BPNN-POP)*. In our proposed model of the Backpropagation Neural Network with popularity (BPNN-POP), we used three inputs; two inputs represented the number of sold items of the previous two issues and the third input represented the popularity of titles and headlines for the target issue, as demonstrated in Figure 4. In this model, we used one hidden layer. After using the training set in the BPNN, the testing set was used to get the predicted values from two models.

*Hybrid ARIMA with BPNN-POP (Hybrid-POP)*. Our proposed model adopted the hybrid model for forecasting sales. In the hybridization, the ARIMA was used to forecast number of items of the target issue by using the number of sold items of the previous two issues. Predicted numbers of items from the ARIMA were used as input for the BPNN-POP along with number of sold items from the previous two issues and the popularity of target issue, as shown in Figure 5. The patterns of the linear and the nonlinear data time series can be captured using this hybrid model.

### 4.2. Experiment Results

**Figure 7:**
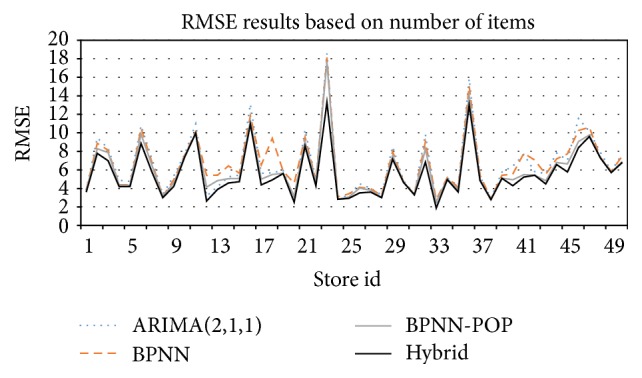
RMSE (by number of items) of 50 stores for ARIMA, BPNN-POP, and Hybrid-POP.

In the experiment, we implemented Hybrid-POP and BPNN-POP methods as our proposed models using popularity as the main input. Other methods, such as Autoregressive Integrated Moving Average, ARIMA(2,1, 1) using historical sales data and BPNN with historical sales data without popularity, were used as benchmarks. Root Mean Square Error was used as an evaluation metric to compare the accuracy of each method.  Figure 7 shows the RMSEs of 50 stores (horizontal axis) for different forecasting methods, where the RMSE of each store is calculated based on the actual numbers of sold items and predicted numbers of items. From the figure, the BPNNs were lower than for the ARIMA for most of the stores in our dataset. Some stores had lower RMSEs using the ARIMA. Due to the nature of data and its fluctuations, it is difficult to judge BPNN or ARIMA as fit models for our data. After using the BPNN-POP, the RMSEs became lower than the RMSEs of the BPNN and the ARIMA for the stores in our dataset. The RMSEs for Hybrid-POP were the lowest when compared to other methods. The forecasting accuracy was improved when we used historical number of sold items data, popularity, and the predicted number of items from the ARIMA forecasting model as inputs for our Hybrid-POP.

#### 4.2.1. The Effects of Popularity

Figure 7 shows that using the popularity of magazine title words as an input for the BPNN-POP and Hybrid-POP helps to enhance forecasting accuracy by reducing the RMSE and making it lower than the ARIMA and BPNN. On the other hand, when ARIMA is used, the RMSEs for some stores in our dataset were lower than in the BPNN while others were higher, as shown in Figure 7. However, after using popularity as an input for BPNN-POP, the RMSEs became lower for all the stores. This implies that popularity enhances the accuracy of forecasting, and it indicates that attractive titles and headlines in magazines have an effect on buyer preferences for these publications. Therefore, the popularity of title words can be used to choose the most appropriate titles and headlines to increase sales and benefits.

#### 4.2.2. The Effects of Using the Hybrid Approach

**Figure 8:**
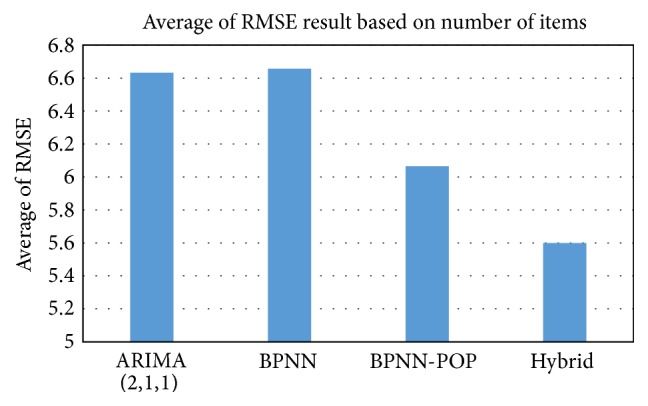
Average of RMSE (by number of items) for ARIMA(2,1, 1), BPNN, BPNN-POP, and Hybrid-POP.

When using Root Mean Square Error as an evaluation metric, as we show in Figure 7, neither the BPNN nor ARIMA fits for all stores in our dataset, due to the nature of some of the data and fluctuations in data. Some stores had linear sales and others nonlinear. In predicting sales, the ARIMA is fit for stores which have linear sales, while neural networks are the best fit for nonlinear stores. The Hybrid-POP model combining the ARIMA and BPNN-POP was used to capture linear patterns and nonlinear patterns of sales for each store. The experiment results are shown in Figure 8. The results represent the average of RMSE for 50 stores in the test set, where the RMSE of each store is calculated based on the actual numbers of sold items and predicted numbers of items.

We note that the RMSE is calculated by considering the residuals of actual number of sold items for each issue and the predicted number of items for that issue, as defined in (6). The maximum number of actual weekly sales (sold items of an issue) of a store is 58. The difference in the average RMSE between BPNN-POP and Hybrid-POP is 0.466 based on the number of sold items. Accordingly, the difference is about 0.8% in percentage, which is important in improving the total sales of stores.

The Hybrid-POP performed better than the other forecasting methods in our experiment. This implies that the Hybrid-POP conducts and captures both linear and nonlinear patterns in our data, and it is more appropriate for our data. The Hybrid-POP model helps to overcome judgment on the behavior of data and uses popularity to improve forecasting.

## 5. Discussion

As shown in Figure 8, using popularity of magazine contents makes the BPNN-POP more accurate and overcomes shortcomings of the traditional forecasting methods represented here by ARIMA and the BPNN without popularity. However, there are both linear and nonlinear sales in the stores, so neither model fits our data. The BPNN-POP using popularity enhanced the accuracy and was a better fit for our data than the ARIMA and BPNN.

The ARIMA model was used to capture the linear pattern in our dataset, and Backpropagation Neural Networks were used to capture the nonlinear pattern. For some stores, using the ARIMA model could be better than using the BPNN, or vice versa. We used the Hybrid-POP to improve the forecasting results by combining the ARIMA and BPNN-POP models to deal with the many potential influencing factors, such as sampling model uncertainty and structure changes. Real-world time series are rarely purely linear or nonlinear. Figure 8 shows that our Hybrid-POP presents a superior and reliable alternative to conventional methods. When they are combined, our Hybrid-POP model is superior to both the ARIMA and BPNN in all points and is also better than our BPNN-POP.

## 6. Conclusion

In this paper, a novel hybrid neural network model is proposed for sales forecasting based on the popularity of article titles. In the publishing industry, the popularity of headlines and titles plays a main role in reader decisions to buy or not to buy. Our proposed hybrid BPNN model considers not only historical data but also the popularity of article titles. The information retrieval techniques and Google search engine were used to derive the popularity of article titles. Using content popularity as an input into the Backpropagation Neural Network has added value to the forecasting process and increased accuracy when compared to the conventional forecasting methods. Existing studies have not considered the effects of content popularity on sales. Moreover, our proposed hybrid model uses the prediction output of ARIMA as input for the BPNN and uses the BPNN for forecasting future sales. Combining the ARIMA and BPNN can utilize the advantages of both methods, thus enabling our proposed hybrid model to capture linear and nonlinear patterns in sales data. Our experimental results show that our proposed forecasting method outperforms conventional techniques which do not consider the popularity of title words. These results imply that our proposed hybrid BPNN model considers the popularity of article titles and prediction output of ARIMA can be a more effective approach for sales forecasting.

Magazine sales may be affected by the locations of stores. It is interesting to analyze the sales/locations of stores and the popularity indexes of magazine issues to find which area is positively correlated with content popularity. In our future work, we will classify the stores spatially and integrate our proposed models with the popularity indexes of magazines in particular areas to enhance sales forecasting.
